# Monoclonal gammopathy of undetermined significance in Chinese population: A prospective epidemiological study

**DOI:** 10.1002/hon.2548

**Published:** 2018-10-01

**Authors:** Ling Ma, Shuang Xu, Jianhua Qu, Jian Hou, Yang Wang, Lei Wen, Yang Liu, Ying Kang, Ming Jiang, Weijun Fu, Juan Du, Lin Zhou, Xiaojun Huang, Zhaoxia Zhang, Jin Lu, on behalf of Chinese medical doctor association multiple myeloma professional committee, Academic working group on multiple myeloma of Chinese geriatric medical association

**Affiliations:** ^1^ Peking University People's Hospital Peking University Institute of Hematology Beijing China; ^2^ Department of Hematology Xinjiang Medical University First Affiliated Hospital Xinjiang Uygur Autonomous Region Urumqi City 830054; ^3^ Department of Hematology Second Military Medical University Affiliated Chang Zheng Hospital Shanghai China; ^4^ Medical Research and Biometrics Center, Fuwai Hospital, National Center for Cardiovascular Diseases, Peking Union Medical College Chinese Academy of Medical Science Beijing China; ^5^ Department of Clinical Laboratory center Xinjiang Medical University First Affiliated Hospital Xinjiang Uygur Autonomous Region Urumqi City 830054

**Keywords:** M protein, monoclonal gammopathy of undetermined significance, prevalence

## Abstract

The aim of this study was to identify the prevalence of monoclonal gammopathy of undetermined significance (MGUS) in different groups of age and the clinical features in China. This multicenter prospective study enrolled 1797 health subjects. The overall prevalence of MGUS was 2.73%. The prevalence of different age groups was 1.19% (41‐50 y), 1.16% (51‐60 y), 2.19% (61‐70 y), 3.66% (71‐80 y), and 7.76% (≥81 y). The prevalence of MGUS in male (n = 843) was 2.97%, while the prevalence of MGUS in female (n = 952) was 2.52%, but this difference of the two groups was not statistically significant. As for subtype of MGUS, IgG subtype was 55.1% (27 cases), IgA subtype was 14.3%, and IgM subtype was 12.2%. The M protein of one case became negative after 3 months, and the others remained positive without obvious disease transformation (follow‐up duration: 3‐7 mo). Thus, the prevalence of MGUS in China was similar to that in Mexican Americans, but lower than that in the other Asian country, American Whites, American Blacks, and Africans, and had a trend of increase with age. Male had higher prevalence of MGUS in China. The most common subtype was IgG.

## 1 INTRODUCTION

Monoclonal gammopathy of undetermined significance (MGUS) is a premalignant disorder caused by the monoclonal immunoglobulin or its fragment, which is secreted by cloned B cells or plasma cells, without features of related malignant disorders such as multiple myeloma, Waldenstrom macroglobulinemia, primary amyloidosis, B‐cell lymphoma, or chronic lymphocytic leukemia. Monoclonal gammopathy of undetermined significance is defined by serum monoclonal protein concentration <30 g/L, clonal plasma cells <10% in the bone marrow, and absence of end‐organ damage (eg, anemia, lytic bone lesions, renal insufficiency, or hypercalcemia associated with the proliferation of the clonal plasma cells).^1^

The prevalence of MGUS ranges from 0.7% to 5.8%, and significantly increases with age.^2^, ^3^, ^4^, ^5^, ^6^, ^7^, ^8^, ^9^, ^10^, ^11^, ^12^, ^13^ The prevalence and type of immunoglobulin may be different between different races.^2^, ^3^, ^4^, ^5^, ^6^, ^14^, ^15^ All multiple myeloma (MM) cases were preceded by MGUS.^16^ Lu et al showed that most of newly diagnosed Chinese patients with MM were Durie‐Salmon stage or ISS stage III, with higher tumor load and poorer prognosis.^17^ Therefore, an epidemiological study of MGUS will help us to early diagnose MM.

This is the first multicenter prospective study aiming to identify the prevalence of MGUS in different groups of age, the type of abnormal M protein, and the difference in China mainland.

## 2 METHODS

### 2.1 Population

Subjects with physical examination (age ≥ 40 y) visiting Peking University People's Hospital, Xinjiang Medical University First Affiliated Hospital and Second Military Medical University Affiliated Chang Zheng Hospital from April 2017 to August 2017 and signed informed consent were included in this study. The research was approved by the People's Hospital of Peking University (approval number: 2017PHB005‐01).

### 2.2 Sample and measurement

Firstly, positive samples were screened through serum protein electrophoresis and verified by immunofixation electrophoresis and then referral to the hematologic clinic to collect medical history (ie, family history, hepatitis, and other major diseases history). All subjects completed tests, including blood routine, liver and kidney function, electrolytes, blood lipid, glucose, N‐terminal pro‐brain natriuretic peptide, cardiac trolonin I, serum free light‐chain assays, serum M protein quantitative, urine free light‐chain quantitative or urine M protein quantitative or 24 hours urine protein quantitative, bone marrow examination (ie, morphology, flow immunological typing, fluorescence in situ hybridization [FISH], and chromosome G‐banding), imaging test (ie, cranial pelvis X‐ray, cervical, thoracic, and lumbosacral vertebral magnetic resonance imaging or PET‐CT). Subjects with MM, amyloidosis, POEMS, and lymphoma were excluded. Once subjects were diagnosed as MGUS, they were followed up and examined with blood and urine tests once every 3 months as well as bone marrow puncture once every year. Subsequent follow‐up duration could be extended to 6 months if the M protein was unchanged within 1 year (ie, 10%). If the disease progression was identified in subjects, it should be registered and reported. Imaging examinations were conducted if necessary based on the decision of researchers.

The serum protein electrophoresis was conducted with Sebia Capillarys 2 electrophoresis apparatus. Urine immunofixation electrophoresis (IFE) was conducted through fully automatic agarose gel electrophoresis apparatus (Sebia Hydrasys 2 electrophoresis apparatus, French Sebia company), using five kinds of antiserum for serum IFE (ie, gamma heavy chain γ [IgG], heavy chain α [IgA], heavy chain μ [IgM], anti‐kappa light chain [free or not free], and anti‐lambda light chain [free or not free]) and five antibodies (ie, the triad antibody of heavy chain γ [IgG], heavy chain α [IgA] and heavy chain μ [IgM], the anti‐free kappa light chain, anti‐free lambda light chain, anti‐kappa light chain [free or not free], and anti‐lambda light chain [free or not free]).

### 2.3 Statistical analysis

All statistical analyses were performed with IBM SPSS 22.0 software. Describe analysis was used for the positive rates and classifications of MGUS. Chi‐square test, *t* test, and Fisher exact test were used to compare rates, and the level of significance was set at *P* values <0.05. Based on the prevalence of the South Korea,^18^ the calculated sample size number of 41 to 50 years, 51 to 60 years, 61 to 70 years, 71 to 80 years, and 80 or higher years in China was 299, 295, 292, 315, and 323, while the total sample size was 1524.

## 3 RESULTS

### 3.1 Methodology validation

Data of 1797 health examination populations from a multicenter were collected. Firstly, 321 serum samples were used to screen through capillary electrophoresis and serum immunofixation electrophoresis. Eight cases were verified to be positive by the both methods with the positive rate of 2.49%. Only one case was negative by capillary electrophoresis, but positive by serum immunofixation electrophoresis. For capillary electrophoresis, the miss detection rate was only 0.32% with short test time, which can be used for batch screening. Therefore, capillary electrophoresis was used for follow‐up screening.

### 3.2 Prevalence and distribution

**Figure 1:**
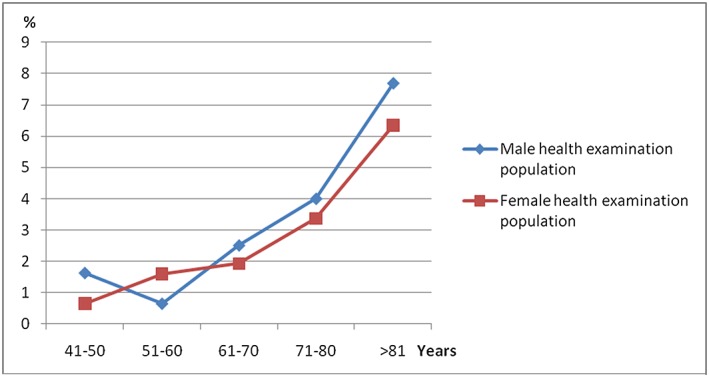
Monoclonal gammopathy of undetermined significance prevalence in patients with different age and gender

**Figure 2:**
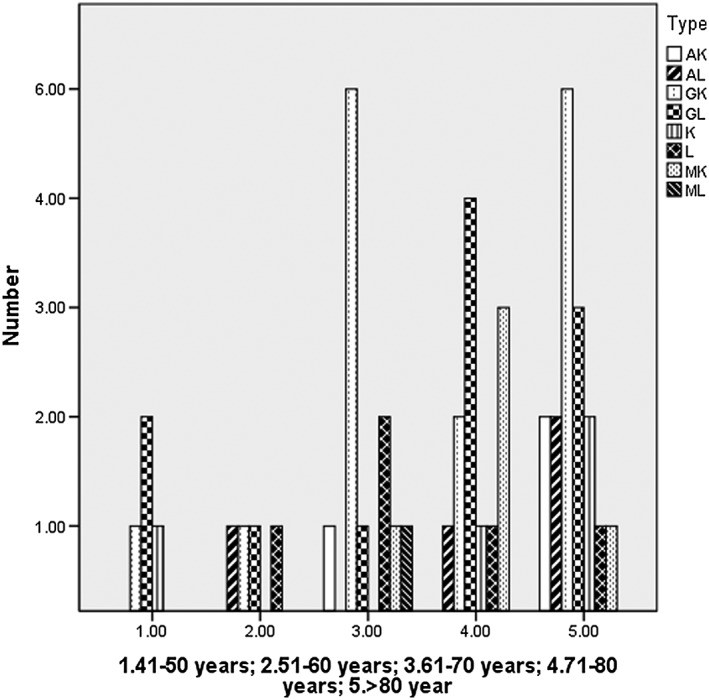
MP types in monoclonal gammopathy of undetermined significance patients in different age groups

Among 1797 cases, 49 cases were diagnosed as MGUS. The overall prevalence was 2.73%. The prevalence in different age groups was 1.19% (41‐50 y), 1.16% (51‐60 y), 2.19% (61‐70 y), 3.66% (71‐80 y), and 7.76% (≥81 y) separately. The prevalence of male cases (n = 843) was 2.97%, while the prevalence of female cases (n = 952) was 2.52%, but this difference of the two groups was not statistically significant (Table 1 and Figure 1). Among 49 cases with evidence of MGUS, 55.1% (n = 27) was IgG subtype, 12.2% was IgM subtype, and 14.3% was IgA subtype. Four cases (8.2%) were kappa light chain subtype and 5 cases (10.2%) were lambda light chain subtype (Figure 2).

**Table 1:** Monoclonal gammopathy of undetermined significance prevalence according to age group and sex

Age, y	Men^a^	Women^a^	*P* value	Total^a^
41‐50	3/183 (1.63)	1/153 (0.65)	0.745	4/336 (1.19)
51‐60	1/154 (0.65)	3/188 (1.60)	0.630	4/342 (1.16)
61‐70	6/239 (2.51)	6/309 (1.93)	0.652	12/548 (2.19)
71‐80	6/150 (4.00)	6/178 (3.37)	0.770	12/328 (3.66)
>80	9/117 (7.69)	8/126 (6.34)	0.682	17/243 (7.00)
Total	25/843 (2.97)	24/954 (2.52)	0.559	49/1797 (2.73)

a The number was presented as number/total number (%).

### 3.3 Bone marrow

Data from a total of 49 cases that were collected showed that the average of bone marrow plasmacyte were 3.4%. Forty‐one cases received the chromosome G‐banding, and five cases (12.2%) were abnormal, which were 45 (X, −Y); t (6;11) (p11;q23); t (7;11); 47, XXX; 47, XX, +2, respectively. The FISH was done in 35 patients, the positive rate was 22.8% (eight cases), including five cases were IgH rearrangement, one case was 1 q21 amplification, two cases were RB1 deletion and D13S319 deletion (7.5%). In which of the five cases, only one case continued to be done IgH translocations including t (11;14), t (4;14), t (14;16), and was found t (11;14). Real‐time quantitative PCR was used to detect the MAGE‐C1/CT7 gene (reference gene ABL) in 28 patients, the positive rate was 78.6% (median: 0.37%, range: 0.01 to 24.69%).

### 3.4 Follow‐up

The M protein of one case became negative after 3 months, and the others remained positive without obvious disease transformation (follow‐up duration: 3‐7 mo).

## 4 DISCUSSION

Patients with MGUS were always asymptomatic, and the majority of patients were identified with the change of immunoglobulin at annual physical examination or visit due to other illnesses and then diagnosed based on serum protein electrophoresis, serum immunofixation electrophoresis, and free light chain. Since the false dismissal rate was high, MGUS can be easily ignored by the clinicians. Therefore, the actual rate of MGUS was much higher than the data.

The prevalence of MGUS was variable across the world (0.7%‐5.8%),^2^, ^3^, ^4^, ^5^, ^6^, ^7^, ^8^, ^9^, ^10^, ^11^, ^12^, ^13^ which may be associated with the difference of race, location, and environment. The prevalence of MGUS in American blacks^2^, ^3^, ^14^, ^15^ and Africans^4^ was more than twice as whites, while the data in Nagasaki City of Japan^5^ and Thailand^6^ showed that their prevalence of MGUS was similar to the whites. Through the NHANES study, Landgren et al^2^ suggested that in the United States, the prevalence of MGUS was significantly higher in blacks (3.67%) compared with whites (2.33%) or Mexican Americans (1.75%). Recently, Landgren et al^3^ also found that the prevalence of the United States in 40 to 49 age groups was 0.88%, and among the blacks, the whites, and Mexican Americans, the data was 3.26%, 0.53% and 2.2%, respectively. Monoclonal gammopathy of undetermined significance prevalence in Hong Kong^13^ was 0.8%, and most of the subtypes were IgG, but the age of their studying object was 50 to 65 years; it was not comparable with the other previous studies. Our study showed that on the Chinese mainland, the prevalence of MGUS over the age of 50 was 3.08%, and the data in the group of 41 to 50 years was 1.19%. The data were higher than that in Japan and South Korea,^18^ which may be associated with the different detection method. As the positive rate of MGUS by capillary electrophoresis was nearly twice than by agarose gel electrophoresis,^19^ the prevalence in China was similar to that in Mexican Americans, but lower than that in the other Asian country (Japan 2.1%, Thailand 2.3%), American whites(2.33%), American blacks(3.67%), and Africans(5.84%).^2^, ^3^, ^4^, ^5^, ^6^

The positive rate was different by different detection methods. In a large study in Olmsted County, Kyle and his colleagues analyzed more than 75% of residents (50 y or older), using agarose gel electrophoresis test, and identified 3.2% of subjects with MGUS. There was a significant age‐dependent increase with the prevalence among persons aged 70 years or older (up to 5.3%).^7^ Subsequently, Dispenzieri and his colleagues used the free light‐chain assay on a majority of the same people and showed a higher prevalence of MGUS in people aged more than 50 (4.2%).^8^ An Italian study^19^ by means of capillary electrophoresis showed that the prevalence of MGUS increased from 3.2% to 6%, which nearly doubled than that previous report. Our study used capillary electrophoresis and found that the prevalence of MGUS over 40 years old was 2.73%, which was lower than that in Italy. This may be related to ethnic differences.

Our data also verified that the men had a higher prevalence (2.97%‐2.52%) in China, which was consistent with previous report in the United States (2.8%‐2.0%).^2^

The data from Mayo Clinic showed that the most common subtype of MGUS was IgG (69%), followed by IgM type (17%) and IgA type (11%).^7^ However, the IgA type in Korea^18^ was the most common subtype (43%), followed by IgG (29%) and IgM (19%). Our data were similar to that of Mayo Clinic, which showed that the most common subtype of IgG was 55.1%, but followed by IgA type (14.3%). Due to the heavy air pollution in China, there may be different types of distribution, such as the IgA type, which was slightly higher than that in Europe and the United States. The prevalence of IgA nephropathy in China is significantly higher than that of other countries, which may be related to the environmental pollution.^20^ Our study showed that the IgM type was 12.2%, which was lower than that of other countries without significant descending.

There were fewer plasma cells and lower cell proliferation in bone marrow of MGUS; hence, the conventional karyotype analysis was always normal in majority of patients. However, with the emergence of the more sensitive methods, people found that the incidence of chromosomal abnormalities was not uncommon. Bacher et al^21^ found that the abnormality rate of iFISH in 302 patients with MGUS was 56% (median application of 11 probe), among which the incidence of 13q deletion was 22.1%, 17p deletion was 2.2%, and t (11;14), t (4;14), t (14;16) was 18.7%, 1.9%, and 1.1%, respectively. Fonseca et al^22^ confirmed that 46% of MGUS had IgH rearrangement. Our data showed that the abnormal rate by the chromosome G‐banding was 12.2%, and by the iFISH detection, the abnormal rate increased to 22.8%, including positive rate of IgH rearrangement was 14.2%, lower than that Ulrike had reported, which may be related to the less probes in our center currently (five to eight).

The MAGE‐C1/CT7 gene was specific for the malignant plasmacyte diseases. Our center has reported^23^ that the positive rate of MAGE‐C1/CT7 was 88.5% in the newly diagnosis of MM (median: 443.2%). Our study found that the positive rate in patients with MGUS was 78.6%, but the expression level was 0.37%, lower than that of MM patients.

Most of the patients with MGUS were “asymptomatic,” although this could last for a long time and was initially considered as a “benign” monoclonal gammopathy. Monoclonal gammopathy of undetermined significance could evolve into a malignant monoclonal gammopathy in a few years, such as lymphoma and MM. A prospective study^16^ in 2009 identified 71 subjects who developed MM with traceable serum using assays for monoclonal proteins and kappa‐lambda free light chains and more than half of them had monoclonal immunoglobulin abnormalities prior to MM diagnosis. Until now MM was considered to be evolved from MGUS. Therefore, the investigation of MGUS (the early state of MM) would help us understand the pathogenesis of MM. The annual transformation probability of MGUS to active MM was 1% followed by 30% of progression at 25 years of follow‐up. Kyle^24^ reported that among the 1384 cases of MGUS in Mayo Clinic, 11% of patients eventually developed into MM, Non‐Hodgkin's lymphoma, primary amyloidosis, Waldenstrom macroglobulinemia, Chronic lymphocytic leukemia, and plasmacytoma. During long‐term follow‐up, 10% of patients progressed in 10 years later followed by 18% in 20 years, 28% in 30 years later, 36% in 35 years later, and 36% in 40 years later, suggesting that 1% of patients with MGUS developed into malignant disease in each year; 11% of the patients died of MGUS progression. Other studies also found that 12% to 17% of patients had progression at the 10 years of follow‐up followed by 25% to 34% of progression in 20 years.^25^, ^26^, ^27^ In our study, the follow‐up duration was short without progression, but the monoclonal immunoglobulin of one case was missing during follow‐up period. Further study with longer follow‐up time was still warrant to examine MGUS.

In conclusion, the prevalence of MGUS in China was similar to that in Mexican Americans, but lower than that in the other Asian country, American whites, American blacks, and Africans, which had a trend of increase with age. Male had higher prevalence of MGUS in China. The most common subtype was IgG. With the gradual increase of life expectancy in China, the screening and regular monitoring of MGUS were relevant.

## CONFLICT OF INTEREST

All authors declare that they have no conflict of interest.
